# Correction: Lara-Banda, M., et al. Alternative to Nitric Acid Passivation of 15-5 and 17-4PH Stainless Steel Using Electrochemical Techniques. *Materials* 2020, *13*, 2836

**DOI:** 10.3390/ma13235322

**Published:** 2020-11-24

**Authors:** María Lara-Banda, Citlalli Gaona-Tiburcio, Patricia Zambrano-Robledo, Marisol Delgado-E, José A. Cabral-Miramontes, Demetrio Nieves-Mendoza, Erick Maldonado-Bandala, Francisco Estupiñan-López, José G. Chacón-Nava, Facundo Almeraya-Calderón

**Affiliations:** 1 Universidad Autonoma de Nuevo Leon, FIME—Centro de Investigación e Innovación en ingeniería Aeronáutica (CIIIA), Av. Universidad s/n. Ciudad Universitaria, San Nicolás de los Garza, Nuevo León 66455, Mexico; marialarabanda@yahoo.com.mx (M.L.-B.); citlalli.gaonatbr@uanl.edu.mx (C.G.-T.); patricia.zambranor@uanl.edu.mx (P.Z.-R.); marisol__1706@hotmail.com (M.D.-E.); jose.cabralmr@uanl.edu.mx (J.A.C.-M.); francisco.estupinanlp@uanl.edu.mx (F.E.-L.); 2 Facultad de Ingeniería Civil, Universidad Veracruzana, Xalapa, Veracruz 91000, Mexico; dnieves@uv.mx (D.N.-M.); eemalban@gmail.com (E.M.-B.); 3 Centro de Investigación en Materiales Avanzados (CIMAV), Miguel de Cervantes 120, Complejo Industrial Chihuahua, Chihuahua, Chih 31136, Mexico; jose.chacon@cimav.edu.mx

The author wishes to make the following correction to this paper [1]. Due to mislabeling, replace:

**Table 3:** Electrochemical noise parameters at various conditions in 5 wt. % NaCl at 49 °C.

Passivated Agent	Stainless Steel Inoxidable	Time (min)	*Rn* (Ω/cm^2^)	*i_corr_* (mA/cm^2^)	LI	Corrosion Type
Citric acid	15-5PH	60	8.01 × 10^4^	6.49 × 10^4^	0.0862	Mixed
		90	5.00 × 10^5^	1.04 × 10^4^	0.0308	Mixed
	17-4PH	60	5.76 × 10^4^	4.51 × 10^4^	0.2492	Localized
		90	3.27 × 10^5^	1.59 × 10^4^	0.0900	Mixed
Nitric acid	15-5PH	60	2.35 × 10^6^	1.1 × 10^5^	0.1871	Localized
		90	1.51 × 10^6^	1.72 × 10^5^	0.1077	Localized
	17-4PH	60	1.03 × 10^6^	2.52 × 10^5^	0.1485	Localized
		90	1.34 × 10^6^	1.94 × 10^4^	0.1727	Localized

**Table 4:** Potentiodynamic polarization parameters in stainless steels passivated at 49 °C, in 5 wt. % NaCl.

Passivated Agent	Stainless Steel	Time (min)	E_corr_ (mV)	E_pit_ (mV)	*i_corr_* (mA/cm^2^)	C. R. (mm/year)
Citric Acid	15-5PH	60	−323	42	5.26 × 10^5^	5.54 × 10^7^
		90	−266	147	4.50 × 10^5^	4.75 × 10^7^
	17-4PH	60	−335	91	9.22 × 10^5^	9.64 × 10^7^
		90	−360	97	5.38 × 10^5^	5.63 × 10^7^
Nitric Acid	15-5PH	60	−228	467	2.16 × 10^5^	2.28 × 10^7^
		90	−228	765	2.27 × 10^5^	2.39 × 10^7^
	17-4PH	60	−271	439	3.51 × 10^5^	3.67 × 10^7^
		90	−279	323	4.41 × 10^5^	4.61 × 10^7^

with

**Table 3:** Electrochemical noise parameters at various conditions in 5 wt. % NaCl at 49 °C.

Passivated Agent	Stainless Steel	Time (min)	Rn (Ω/cm^2^)	*i_corr_* (mA/cm^2^)	LI	Corrosion Type
Citric acid	15-5PH	60	8.01 × 10^−4^	6.49 × 10^−4^	0.0862	Mixed
		90	5.00 × 10^−5^	1.04 × 10^−4^	0.0308	Mixed
	17-4PH	60	5.76 × 10^−4^	4.51 × 10^−4^	0.2492	Localized
		90	3.27 × 10^−5^	1.59 × 10^−4^	0.0900	Mixed
Nitric acid	15-5PH	60	2.35 × 10^−6^	1.1 × 10^−5^	0.1871	Localized
		90	1.51 × 10^−6^	1.72 × 10^−5^	0.1077	Localized
	17-4PH	60	1.03 × 10^−6^	2.52 × 10^−5^	0.1485	Localized
		90	1.34 × 10^−6^	1.94 × 10^−4^	0.1727	Localized

**Table 4:** Potentiodynamic polarization parameters in stainless steels passivated at 49 °C, in 5 wt. % NaCl.

Passivated Agent	Stainless Steel	Time (min)	E_corr_ (mV)	E_pit_ (mV)	*i_corr_* (mA/cm^2^)	C. R. (mm/year)
Citric Acid	15-5PH	60	−323	42	5.26 × 10^−5^	5.54 × 10^−7^
		90	−266	147	4.50 × 10^−5^	4.75 × 10^−7^
	17-4PH	60	−335	91	9.22 × 10^−5^	9.64 × 10^−7^
		90	−360	97	5.38 × 10^−5^	5.63 × 10^−7^
Nitric Acid	15-5PH	60	−228	467	2.16 × 10^−5^	2.28 × 10^−7^
		90	−228	765	2.27 × 10^−5^	2.39 × 10^−7^
	17-4PH	60	−271	439	3.51 × 10^−5^	3.67 × 10^−7^
		90	−279	323	4.41 × 10^−5^	4.61 × 10^−7^

The authors would like to apologize for any inconvenience caused to the readers by these changes.
